# Economic burden and quality of life of IgG4-related disease in China: A comparative analysis of three treatment regimens

**DOI:** 10.1515/rir-2026-0031

**Published:** 2026-10-22

**Authors:** Yifei Wang, Jialing Jiang, Yuxue Nie, Jingna Li, Jialei Zhang, Qinhuan Luo, Xinli Yang, Nianyi Zhang, Shuo Liu, Jueming Lei, Jiaxin Zhou, Yunyun Fei, Linyi Peng, Wen Zhang

**Affiliations:** Department of Rheumatology and Clinical Immunology, Peking Union Medical College Hospital, Chinese Academy of Medical Sciences & Peking Union Medical College, National Clinical Research Center for Rheumatic and Autoimmune Diseases (NCRC-RAD), State Key Laboratory of Complex Severe and Rare Diseases, The Ministry of Education Key Laboratory, Beijing, China; Department of Rheumatology, Tongji Medical College, Huazhong University of Science and Technology, Wuhan, Hubei Province, China; Department of Basic Medical Science, School of Medicine, Tsinghua University, Beijing, China; Department of Rheumatology, Qingdao Municipal Hospital Group, Qingdao, Shandong Province, China; Department of Planning and Development, Peking Union Medical College Hospital, Beijing, China; National Human Genetic Resource Center, Beijing, China; National Research Institute for Family Planning, Beijing, China

**Keywords:** IgG4-related disease, glucocorticoids, patient reported outcome measures, quality of life, cohort study

## Abstract

**Background and Objectives:**

IgG4-related disease (IgG4-RD) is a chronic fibroinflammatory disorder requiring prolonged treatment, placing substantial financial burden on patients. This study compares the direct medical costs of three treatment regimens—glucocorticoid (GC), GC combined with Mycophenolate mofetil (MMF), and rituximab (RTX) —and evaluates the patients’ health-related quality of life (HR-QoL) using the EuroQol 5-Dimension 5-Level scale (EQ-5D-5 L) to assess cost-effectiveness.

**Methods:**

Our retrospective cohort study consecutively distributed EQ-5D-5 L questionnaires to treatment-naïve IgG4-RD outpatients. Patients formed three cohorts: 30 with GC, 30 with GC+MMF, and 20 with RTX. Patients on other regimens were excluded. HR-QoL was assessed using EQ-5D-5 L questionnaires and visual analog scales. Quality-adjusted life years (QALYs) were calculated, and incremental cost-effectiveness ratios were computed. Probabilistic sensitivity analysis with Monte Carlo simulations evaluated cost-effectiveness across willingness-to-pay thresholds.

**Results:**

First-year treatment costs were $1284.53 for GC, $2090.96 for GC+MMF, and $6175.97 for RTX. RTX group demonstrated the lowest relapse rate. No significant differences were observed in HR-QoL scores among groups, though visual analog scale (VAS) scores favored GC over GC+MMF. Median incremental cost-effectiveness ratios of probabilistic sensitivity analysis were $13,310/QALY for RTX versus GC and $17,412/QALY for RTX versus GC+MMF. GC demonstrated highest cost-effectiveness probability at willingness-to-pay thresholds below $15,000/QALY. Inverse probability of treatment weighting analysis confirmed these findings’ robustness after controlling for selection bias.

**Conclusions:**

While RTX offers superior efficacy with lowest relapse rates, its high price leads to limited usage in China. GC-based regimens provide reasonable outcomes at lower costs, making GC or GC+MMF more economical choices.

## Introduction

IgG4-related disease (IgG4-RD) is a chronic fibroinflammatory disorder with multi-organ involvement, elevated serum IgG4 concentrations, and IgG4-positive plasma cells infiltrating tissues.^[1]^ The estimated incidence of IgG4-RD in Japan and USA was 0.28–1.08 and 1.39 per 100,000 person-years.^[2,3]^ Typically affecting older adults,^[4]^ IgG4-RD frequently relapse,^[5]^ necessitating long-term treatment and monitoring, which imposes a substantial financial strain on patients.

Glucocorticoid (GC) is the first-line treatment for IgG4-RD.^[6]^ Combining GC with immunosuppressants like mycophenolate mofetil (MMF) improves outcomes during the maintenance stage,^[7, 8, 9]^ while rituximab (RTX) serves as steroid-sparing therapy, particularly for steroid-resistant and refractory cases.^[10,11]^ Since only GC has formal indication for IgG4-RD currently, other agents lack insurance coverage in China, creating distinct treatment patterns compared to Western countries.

Despite growing clinical recognition, the socioeconomic impact of IgG4-RD remains poorly understood. Only one study by Wallace *et al*., has quantified IgG4-RD-associated health-care expenditures in an American cohort, yet without comparing treatments.^[12]^ Notably, no economic evaluations exist in Asian populations, where reimbursement policies shape distinct treatment paradigms. These disparities highlight the need for region-specific economic evaluation incorporating local therapeutic patterns and patient-reported outcomes.

To address this gap, we focused on three globally relevant regimens: GC, the first-line therapy endorsed by international guidelines; GC combined with MMF, a cost-effective strategy widely adopted in Chinese clinical practice; and RTX, a biologic agent increasingly utilized in China despite insurance barriers, reflecting its established role in Western treatment algorithms.

In this study, we compared the direct medical costs (DMC) among three treatment groups: GC, GC combined with MMF, and RTX. Additionally, we assessed the Health-Related Quality of Life (HR-QoL) and Quality-Adjusted Life Years (QALYs) using the EuroQol 5-Dimension 5-Level scale (EQ-5D-5 L) to evaluate these treatment approaches from the patients’ perspective. Sensitivity analyses were also conducted to assess the robustness of the economic findings under varying assumptions and parameter uncertainties. Understanding the economic burden can assist clinicians in communicating with patients and choosing more suitable treatment regimens, fostering the balance among clinical efficacy, financial sustainability, and quality of life.

## Materials and Methods

### Patient Recruitment and Data Collection

This study included IgG4-RD patients who visited the outpatient clinic, Department of Rheumatology, Peking Union Medical College Hospital, from 2020 to 2024 and who completed a regular follow up plan. Patients were diagnosed using the 2019 ACR/EULAR or 2020 diagnostic criteria.^[13,14]^ Those with severe organ damage, malignancy, or active infections were excluded. Relapse was defined as a recurrence of symptoms and/or worsening of imaging findings or new organ involvement, with any item of IgG4-RD RI ≥ 2. Disease relapse was systematically evaluated at each follow-up visit (typically at 3, 6, 12, 18, and 24 months after treatment initiation).

Between September and December 2024, EQ-5D-5 L questionnaires were distributed to the patients from the same outpatient clinic. The study period encompassed patients who initiated treatment since January 2020, with follow-up extending until the questionnaire distribution date. The endpoint of the research period was defined as the date of the last clinical visit or December 2024, whichever came first. Eligible questionnaires were screened, and medical records were reviewed. After screening for eligibility and reviewing medical records, the study population naturally consisted of patients receiving one of the three most common treatment regimens in our clinical practice: 30 patients treated with GC, 30 with GC+MMF, and 20 with RTX naïve to GC/MMF. To reflect real-world treatment patterns, we excluded a small number of patients receiving other regimens that did not comply with standardized clinical guidelines or recommendations. Baseline characteristics were compared across groups to confirm comparability. To address potential selection bias in treatment assignment, we conducted an inverse probability of treatment weighting (IPTW) sensitivity analysis. Propensity scores were estimated using multinomial logistic regression with baseline covariates including age, gender, number of organs involved, IgG level, and IgG4 level. We extracted baseline data and health costs from medical records. DMC included costs of medical service, laboratory tests, pathological examination and surgery, imaging tests, IgG4-RD medications, complication prevention drugs, adverse effect treatments (infection, steroid-induced diabetes, steroid-induced hypertension), and others (inpatient-related costs). All costs were converted to USD using an exchange rate of 7.29 (CNY/USD).

### Evaluation of HR-QoL and QALYs

The EQ-5D-5 L questionnaire gathers data across five dimensions affecting patients due to disease: mobility, self-care, daily activities, pain/discomfort, and anxiety/depression. Each dimension includes five levels of severity (no/ slight/moderate/severe/extreme problems). Patients selected the statement that best reflected the health status of the past year during their latest outpatient visit. The final EQ-5D-5 L index of each patient was calculated with five answers, ranging from-0.654 (indicating poor QoL) to 1 (indicating good QoL), using the Chinese value set.

Information about HR-QoL was collected using the EQ-5D-5 L and the visual analog scale (VAS), with permission from the EuroQol Research Foundation (Registration ID: 72898). These two tools have wide application in studying the economic burdens of different diseases.^[15,16]^

The VAS is a vertical scale ranging from 0 (worst health) to 100 (best health), assessing patient’s health perception. QALYs represent the product of HR-QoL and time until death or significant health deterioration. Given IgG4-RD’s chronic course with low mortality,^[17, 18, 19]^ we assumed stable health states during the 1-year follow-up, using baseline HR-QoL for QALY estimation. The primary purpose of assessing HR-QoL and deriving QALYs was to facilitate the subsequent cost-utility analysis evaluating the economic efficiency of the different treatment regimens.

### Economic Evaluation Analysis

Incremental Cost-Effectiveness Ratio (ICER): The primary outcome of the cost-utility analysis was the ICER. ICERs were calculated pairwise using the following formula (1):


(1) $$ICER=\frac{{\text{ Cost }}_{\text{TreatmentB }}-{\text{ Cost }}_{\text{TreatmentA }}}{\left({\text{ QALY }}_{\text{TreatmentB }}-{\text{ QALY }}_{\text{TreatmentA }}\right)}$$

Probabilistic Sensitivity Analysis (PSA): We performed 10, 000-iteration Monte Carlo PSA simulations to assess parameter uncertainty.^[20]^ Key clinical and economic parameters were assigned probability distributions reflecting their inherent uncertainty: direct medical costs were modeled using gamma distributions characterized by sample-specific means and standard errors to preserve non-negative and right-skewed properties; health utility estimates (QALYs) followed beta distributions parameterized by observed utility scores and sample sizes to constrain values within the 0–1 interval; the QALY penalty per relapse event was represented by a triangular distribution with minimum = 0.100, mode = 0.125, and maximum = 0.150 based on clinical expert consensus;^[21]^ relapse duration adopted a triangular distribution (min = 0.25 months, mode = 0.375 months, max = 0.5 months) informed by disease patterns; annual relapse counts were modeled *via* Poisson distributions with group-specific event rates (λ) derived from empirical data.

Cost-Effectiveness Acceptability Curve: Cost-Effectiveness Acceptability Curves were constructed to visualize the probability of each treatment strategy being cost-effective across a spectrum of societal willingness-to-pay (WTP) thresholds per additional QALY gained. These curves were generated by calculating, for each WTP value, the proportion of PSA iterations in which a given therapeutic strategy yielded the highest net monetary benefit. The net monetary benefit was computed as formula (2):


(2)  Netmonetarybenefit =( Effectiveness  ×  WTP )− Cost 

Final cost-effectiveness probabilities were specifically reported at China’s 2024 per capita gross domestic product (GDP) reference thresholds (1 × and 3 × GDP), which served as key decision-making benchmarks for interpreting the economic viability of interventions in the local healthcare context. According to the National Bureau of Statistics of China,^[22]^ the 2024 per capita GDP was 95,749 CNY (13,134.29 USD). An ICER below the lower bound (1 x GDP) is generally considered highly cost-effective, while an ICER below the upper bound (3 x GDP) is typically regarded as cost-effective within the Chinese healthcare setting.

### Statistical Analysis

Baseline characteristics were summarized as follows: categorical variables presented as frequencies (percentages), and continuous variables reported as mean ± standard deviation when normally distributed or median with interquartile range (IQR) for non-normally distributed variables. Group comparisons for continuous variables used the Kruskal-Wallis test, with statistical significance defined by a two-sided *P*-value < 0.05. All statistical analyses were performed using IBM^®^ SPSS^®^ (version 27; Armonk, NY, USA) and R (version 4.2.1; R Foundation for Statistical Computing, Vienna, Austria), with figures generated in GraphPad Prism (version 8.0.0; San Diego, CA, USA).

## Results

### Baseline Characteristics of Demographic and Clinical Features

Baseline characteristics showed male predominance (66.7%–70.0%) across groups, with diagnosis typically in the fifties. No significant differences existed in affected organ numbers or IgG4-RD RI scores among groups (Table 1). Patients in the RTX group had higher serum IgG levels (*P* = 0.028), higher serum IgG4 levels (*P* = 0.020).

**Table 1:** Baseline characteristics of IgG4-RD patients among three treatment groups

Characteristics	GC (*n* = 30)	GC+MMF (*n* = 30)	RTX (*n* = 20)	*P* value
Demographic and clinical features				
Gender (male, *n*%)	20, 66.7%	20, 66.7%	14, 70%	0.963
Age of diagnosis (median, IQR) (years)	56 (49-65)	55 (49-60)	56 (49-61)	0.784
Follow-up duration (months, median, IQR)	14 (11-24)	13 (11-16)	14 (9-23)	0.452
IgG4-RD responder index scores (median, IQR)	6 (4-8)	6 (4.5-10)	6 (4-12)	0.697
Number of organs involved (median, IQR)	3 (2-4.75)	4 (3-5)	3.5 (2.75-6)	0.645
Serologic features (median, IQR)				
Estimated sedimentation rate (mm/h)	17.50 (13.50-29.25)	23.50 (14.00-50.50)	49.00 (15.50-73.00)	0.196
Hypersensitive C-reactive protein (mg/L)	1.98 (1.02-5.07)	1.98 (0.75-3.51)	2.00 (0.64-4.19)	0.987
IgG (g/L)	19.86 (14.45-27.91)	18.07 (15.19-22.63)	27.33 (17.57-31.69)	0.028
IgE (kU/L)	235.00 (50.65-1018.00)	247.50 (98.85-538.80)	349.50 (186.00-633.50)	0.510
IgG4 (mg/L)	8879 (2726-16050)	4658 (2307-12074)	21456 (5526-29881)	0.020
Complement 3 (g/L)	0.94 (0.72-1.12)	0.99 (0.90-1.07)	0.89 (0.61-1.07)	0.269
Complement 4 (g/L)	0.17 (0.09-0.20)	0.17 (0.14-0.25)	0.14 (0.07-0.23)	0.218

GC, Glucocorticoid; MMF, mycophenolate mofetil; RTX, rituximab.

Regarding follow-up duration, 55 patients (68.9%) completed at least 12 months of follow-up. Among these, 13 patients (16.3%) had follow-up duration ≥ 24 months: eight in the GC group and five in the RTX group. The median follow-up duration was 14 months (IQR: 11–24) for GC, 13 months (IQR: 11–16) for GC+MMF, and 14 months (IQR: 9–23) for RTX (Table 1).

### Description of Costs and Fee Categories

As shown in Table 2, DMC were lowest in the GC group and highest in the RTX group across most categories. Median imaging costs were comparable due to uniform follow-up computed tomography protocols. Preventive costs for complications were elevated in GC and GC+MMF groups, with one steroid-induced diabetes case observed exclusively in the GC cohort.

**Table 2:** Monthly median costs of different categories among three groups.

USD^a^	GC (*n* = 30)	GC+MMF (*n* = 30)	RTX (*n* = 20)
Medical service	7.94 (5.45-15.74)	10.91 (6.98-16.09)	14.95 (6.85-20.82)
Laboratory tests	31.13 (19.88-50.24)	45.83 (29.55-53.73)	133.87 (126.78-239.53)
Pathological examination / Surgery	0 (0-6.86)	0 (0-9.86)	0.70 (0-48.19)
Imaging tests	31.21 (18.76-52.85)	31.65 (19.50-55.04)	40.30 (24.40-110.56)
Drugs for IgG4-RD	22.69 (3.20-46.15)	33.73 (25.97-54.66)	341.98 (196.50-477.51)
Drugs for prevention of complications	4.40 (3.11-11.26)	6.43 (3.16-11.86)	3.91 (1.31-11.69)
Others^b^	0 (0-0)	0 (0-0)	20.71 (6.10-99.79)

a The conversion between USD and CNY was based on the exchange rate of 7.29 as of February 10, 2025.

b Inpatient-related costs, including hospitalization for RTX infusion. GC, Glucocorticoid; MMF, mycophenolate mofetil; RTX, rituximab; IgG4-RD, IgG4-related disease.

Drug costs dominated expenses across all groups, particularly in the RTX cohort (Figure 1A). First-year expenses were $1284.53 for GC, $2090.96 for GC+MMF, and $6175.97 for RTX. For patients with more than 2 years of follow-up, 2nd year expenses declined substantially. The RTX group had the highest accumulation expenses, followed by the GC+MMF group and GC group (Figure 1B). Moreover, the RTX group showed the lowest relapse rate (0/20) compared with the GC group (8/30) and the GC+MMF group (4/30).

**Figure 1:**
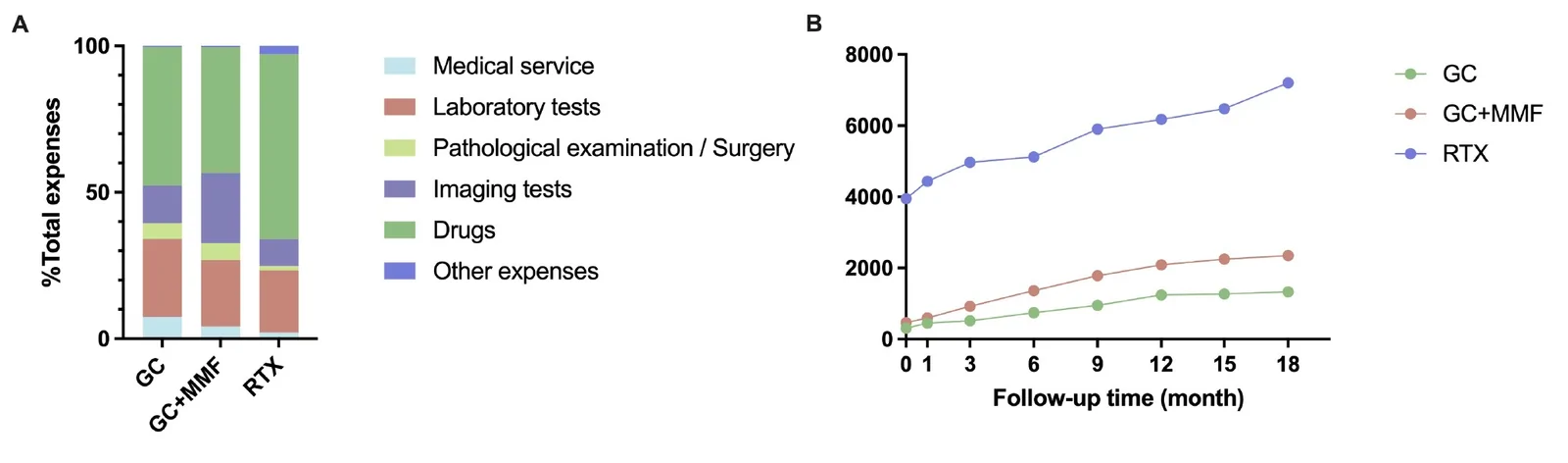
Details of medical costs among three groups. (A) Percentage of different fee categories in total expenses; (B) Accumulation of total expenses. GC, Glucocorticoid; MMF, mycophenolate mofetil; RTX, rituximab.

### Cost-effectiveness Evaluation: QALY and ICER analysis

Our analysis of EQ-5D dimensions revealed that IgG4-RD patients across all treatment groups maintained good functional capacity in mobility, self-care, and usual activities (Figure 2A). However, pain/discomfort was reported by 26.7% (GC), 46.7% (GC+MMF), and 30% (RTX); anxiety/depression by 13.3%, 30%, and 20%, respectively.

**Figure 2:**
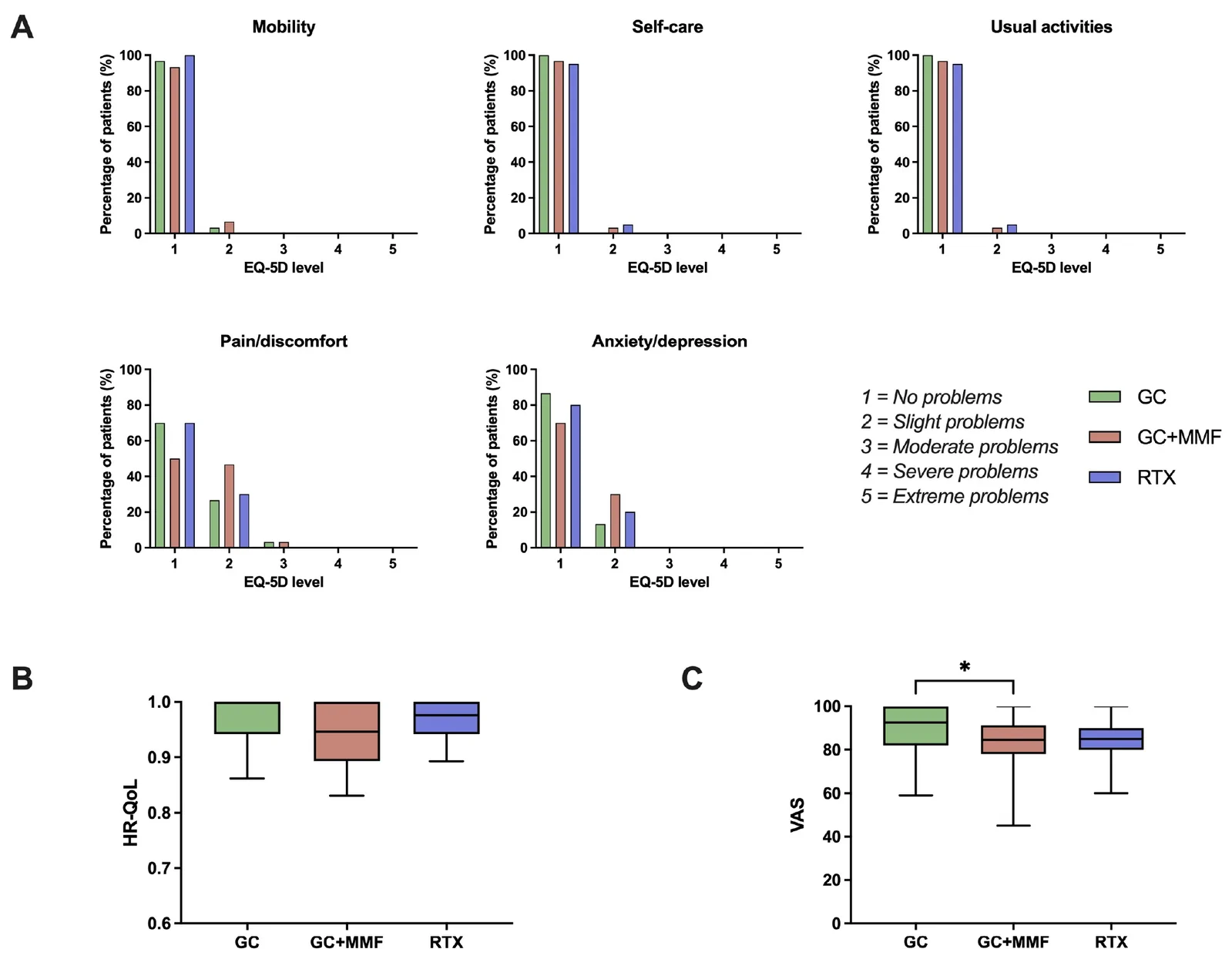
EQ-5D dimension distributions and HR-QoL/VAS score comparisons among treatment groups. (A) Stacked bar charts showing the severity distributions of the five EuroQol-5D dimensions across treatment groups; (B) Box plots of EQ-5D health-related quality of life index scores; (C) Box plots of EQ-VAS scores. GC, Glucocorticoid; MMF, mycophenolate mofetil; RTX, rituximab; HR-QoL, health-related quality of life; VAS, visual analog scale.

No statistically significant differences emerged in overall HR-QoL between groups (*P* = 0.117, Figure 2B), though VAS scores demonstrated significant variation (*P* = 0.039), with post-hoc analysis confirming higher values for GC versus GC+MMF (adjusted *P* = 0.049, Figure 2C).

The cost-effectiveness evaluation yielded distinct patterns depending on the analytical approach (Table 3). After IPTW adjustment, annual treatment costs were $1300.64 for GC, $2308.46 for GC+MMF, and $6, 202.77 for RTX. These findings were consistent with the main analysis, confirming the robustness of our cost-effectiveness conclusions despite controlling for potential confounding factors. All relapse-adjusted ICER values substantially exceeded China’s accepted willingness-to-pay thresholds (13,134.29–39,402.88 USD/ QALY based on 2024 per capita GDP), indicating limited cost-effectiveness for both GC+MMF and RTX groups considering relapse-related quality adjustments.

**Table 3:** Incremental cost-effectiveness ratios of treatment strategies for IgG4-RD.

Comparison	Incremental Cost (USD)	Incremental QALYs	ICER (Cost per QALY)
Base-case Analysis			
GC+MMF *vs*. GC	806.43	-0.032	-25,272.45
RTX *vs*. GC	4891.44	-0.003	-1603051.79
RTX *vs*. GC+MMF	4085.01	0.029	141554.86
Relapse-Adjusted Analysis^a^			
GC+MMF *vs*. GC	806.43	-0.026	-31439.55
RTX *vs*. GC	4891.44	0.009	515851.45
RTX *vs*. GC+MMF	4085.01	0.035	116302.31
IPTW-Adjusted Analysis^b^			
GC+MMF *vs*. GC	1007.82	-0.031	-32933.00
RTX *vs*. GC	4902.13	-0.001	-7172126.00
RTX *vs*. GC+MMF	3894.31	0.030	130165.00

a Relapse-adjusted analysis reflects QALY adjustments based on disease relapse rates.

b IPTW-adjusted analysis accounts for potential selection bias using inverse probability of treatment weighting based on baseline covariates. QALYs, quality-adjusted life years; ICER, incremental cost-effectiveness ratio; GC, Glucocorticoid; MMF, mycophenolate mofetil; RTX, rituximab; IPTW, inverse probability of treatment weighting.

The annual expenses for IgG4-RD patients in this cohort in the first year were $1284.53 for GC, $2090.96 for GC+MMF, and $6175.97 for RTX.

### Sensitivity Analysis

Probabilistic sensitivity analysis revealed significant variation in cost-effectiveness profiles among treatment strategies. The median incremental cost-effectiveness ratios were 3, 635 USD/QALY for GC+MMF versus GC (IQR: -1069 to 10,192), 13, 310 USD/QALY for RTX versus GC (IQR: 8006 to 21,252), and 17, 412 USD/QALY for RTX versus GC+MMF (IQR: 7890 to 33,278). After IPTW adjustment for potential selection bias, these values remained largely consistent at 3242.80, 12,482.85, and 16,474.62 USD/QALY respectively, confirming the robustness of our cost-effectiveness findings despite potential treatment assignment bias.

The differences between these probabilistic estimates and the deterministic ICERs in Table 3 reflect distinct ways of handling parameter uncertainty. The deterministic analysis applies fixed point estimates for all parameters, including relapse penalties, which can amplify differences when one strategy has zero events (RTX: 0/20 relapses) compared to others with higher rates (GC: 8/30). In contrast, PSA incorporates uncertainty by sampling from probability distributions, allowing relapse events and their associated utility decrements to vary across simulations. Therefore, PSA results represent the central tendency across plausible scenarios, while deterministic results reflect outcomes under fixed assumptions.

The probabilistic sensitivity analysis evaluated the cost-effectiveness of three treatment strategies. After adjustment with QALY penalty per relapse event, Figure 3. A depicts GC as the least costly and least effective (< 0.50 QALYs), GC+MMF as intermediate in cost and effectiveness (0.50–1.00 QALYs), and RTX as the most effective (> 0.75 QALYs) but also the most costly, with 95% confidence ellipses representing variability. Arrows denote incremental cost and QALY gains relative to GC. As shown in Figure 3. B, we compared GC+MMF versus GC (mean ICER: $6836.02/QALY) and RTX versus GC (mean ICER: $13,768.88/QALY), with most points in the northeast quadrant, indicating higher costs and greater effectiveness. Figure 3. C demonstrates that at low WTP thresholds, GC is most likely cost-effective, whereas GC+MMF achieves a 70% probability at $15,000/QALY. At WTP thresholds ≥ $20,000/QALY, RTX’s probability rises to approximately 75%, surpassing or nearing GC+MMF. These findings illustrate key trade-offs: GC is favored at low WTP, GC+MMF performs robustly at moderate thresholds, and RTX becomes preferable at higher WTP levels.

**Figure 3:**
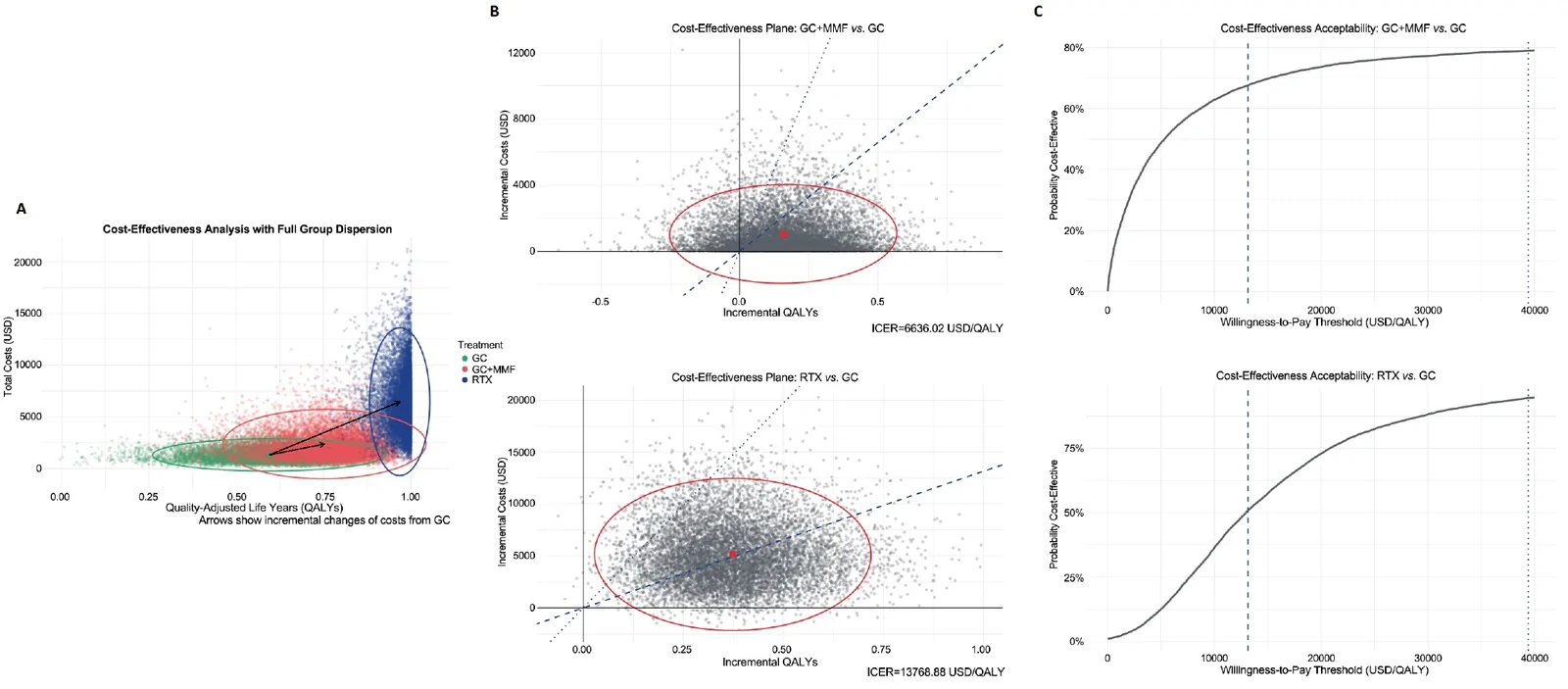
Cost-effectiveness analysis of three IgG4-RD treatment regimens. (A) Cost-effectiveness plane showing mean costs and QALYs with 95% confidence ellipses for GC, GC+MMF, and RTX treatments. (B) Incremental cost-effectiveness scatter plot comparing treatment strategies. (C) Cost-effectiveness acceptability curves showing probability of cost-effectiveness across WTP thresholds. IgG4-RD, IgG4-related disease; QALY, quality-adjusted life year; WTP, Willingness-to-pay.

## Discussion

This is the first economic and quality-of-life study of IgG4-RD treatments in China. By comparing three main treatment approaches, we aimed to inform clinical decision-making. While RTX offers superior efficacy with lowest relapse rates, its high price leads to limited usage in China. GC-based regimens provide reasonable outcomes at lower costs, making GC or GC+MMF more economical choices. Our findings help patients and clinicians achieve a balance between clinical efficacy, financial sustainability, and quality of life.

### Cost Analysis and International Comparisons

The annual expenses for IgG4-RD patients in this cohort in the first year were $1284.53 for GC, $2090.96 for GC+MMF, and $6175.97 for RTX. Till now, there exists no standardized treatment guidelines internationally, making the DMC in IgG4-RD management deviated across different countries. Wallace *et al*., reported that patients with IgG4-RD from the US incurred significantly higher pharmacy costs than the general population, averaging $10,817.13 during the first-year follow-up in 2024, with the higher utilization of biologic therapies, such as RTX.^[12]^ Higher costs in the US possibly reflect differences in treatment protocols and healthcare systems. First, the dosing scheme of RTX between US and China is different, with US RTX dosing including initial induction (1 g days 1 & 14) plus 6-month maintenance (1 g),^[23,24]^ whereas China uses only 6-month maintenance (1 g) without induction. Moreover, differences in insurance coverage may drive divergent treatment selection patterns: in the US, broader reimbursement of biologic therapies enables more liberal RTX use across a wider spectrum of disease severity, as reflected in RTX utilization rates of 8.8% in US cohort,^[12,25]^ whereas in China, the absence of insurance coverage for RTX leads the majority of patients to choose GC-based regimens, with an initiation rate of RTX less than 1.0%.^[26]^

### Longitudinal Cost Patterns and Relapse Outcomes

Our longitudinal cost analysis demonstrated significant reductions in treatment expenses from the first to second year across all groups. For patients with more than 2 years of follow-up among the GC group, GC+MMF group, and RTX group, annual expenses for the 1st year were 4.5, 9.2, and 5.2 times higher than those for the 2nd year, respectively, which was possibly caused by the relatively stable disease state and decreased frequency of visits in the second year. Till the last visit of follow-up, patients with RTX exhibited the lowest relapse rate compared with the other groups,^[24]^ which aligns with other research, making RTX an ideal choice for patients with refractory IgG4-RD.^[27]^

### Quality of Life Assessment

Patient-reported outcomes are also crucial for assessing treatment value.^[28]^ Our EQ-5D analysis demonstrated that patients across all treatment groups maintained functional capacity in mobility, self-care, and usual activities, indicating preserved basic functioning regardless of treatment approach. However, the quality-of-life analysis revealed that patients from the GC+MMF group reported greater impairment, especially in the dimensions of pain/discomfort and anxiety/ depression.

Despite these dimensional differences, overall HR-QoL scores showed no differences among those three groups. In contrast, VAS scores demonstrated significant variation among groups, with the GC group exhibiting the highest VAS scores.

This may be attributed to several factors. First, with a median follow-up of approximately 14 months, GC-related adverse effects such as osteoporosis, steroid-induced diabetes, and cataracts may not yet have manifested, potentially masking a long-term quality-of-life advantage of RTX. Second, the EQ-5D-5 L instrument primarily captures functional domains and may be insufficiently sensitive for IgG4-RD patients whose basic daily functioning is generally well-preserved, as further evidenced by its discordance with VAS scores.^[29]^ Third, the high out-of-pocket cost of RTX may impose financial stress that counteracts the benefit of superior disease control, consistent with evidence that financial burden negatively affects patient-reported wellbeing.^[30]^ Fourth, the immediate symptom relief from GC may be more perceptible to patients than the delayed, preventive benefit of RTX.^[31]^

### Cost-effectiveness Implications

The cost-effectiveness analysis provided crucial insights for healthcare resource reallocation.^[32]^ The GC group demonstrated the highest probability of cost-effectiveness (exceeding 70%) at WTP thresholds below $15,000 (China’s 1 x GDP) per QALY. The GC+MMF group demonstrated a lower relapse rate compared to GC alone,^[33]^ while maintaining cost advantages over RTX-based regimens. RTX approached over 75% probability of cost-effectiveness only at WTP values substantially exceeding China’s 3x GDP threshold, specifically above $20,000 per QALY.

These findings should be complied with the real-world scenarios: no drugs are approved for IgG4-RD,^[34]^ resulting in off-label use of immunosuppressants and RTX without insurance coverage. Although RTX exhibits the lowest recurrence rates, patient’s access to RTX is restricted by its high cost ($1087.65 per vial). This economic reality of health insurance possibly be the reason that 86.2% of patients in other Chinese cohorts receive GC, immunosuppressants, or combination therapy, while less than 1.0% initially receive RTX^[26]^ —contrasting sharply with the approximately 8.8% of US patients and 21.2% of French patients who receive RTX therapy.^[25,35]^

The IPTW sensitivity analysis strengthens our findings by demonstrating that the cost-effectiveness patterns remain consistent even after adjusting for potential selection bias in treatment assignment. The substantial reduction in absolute cost values after IPTW weighting reflects the importance of controlling for patient characteristics that influence both treatment selection and outcomes. Notably, the relative cost-effectiveness rankings remained unchanged.

### Clinical Applications and Policy Considerations

The efficacy and adverse effects of GC in treating IgG4-RD are well-documented. Martina *et al*., reported that in the second year of GC treatment, the cumulative incidence rates of osteoporosis, diabetes, and cataracts among patients with systemic lupus erythematosus were 2%, 2%, and 6%, respectively.^[36]^ The gradual onset of these symptoms during the initial treatment may explain the transient improvement in HR-QoL and VAS scores observed in the GC group of our study.^[37]^ Compared to GC and immuno-suppressants, long-term RTX treatment is generally well tolerated, with no cumulative adverse effects reported.^[37,38]^ These factors make RTX a preferable option for maintenance therapy.

These findings offer potential strategies for clinical guideline development and policy-making. Coherently, clinical decision-making frameworks should involve collaborative discussions, incorporating factors including clinical efficacy, patients’ willingness and ability to pay, and long-term QALYs.

For regions with well-supportive insurance system, given the lower recurrence rate of RTX and its potential to reduce long-term complications, incorporating cost-effectiveness evaluations of RTX into reimbursement discussions could be helpful. Considering the lower medical costs and satisfactory life quality in the GC and GC+MMF groups, resource-limited settings may prefer GC-based regimens.

### Study Limitations

Our study has several limitations. First, given the limited number of patients with ≥ 24 months of follow-up, a formal multi-year cost analysis adjusted for treatment duration was not feasible; future studies with larger sample sizes and extended follow-up are warranted. Second, as a tertiary referral center, potential selection bias toward complex cases and limited sample size restricted generalizability. Although serum IgG and IgG4 levels were higher in the RTX group, clinical disease activity indicators, including RI scores and the number of organs involved, showed no significant intergroup differences, and IPTW analysis further adjusted for these serological imbalances; nonetheless, residual confounding cannot be entirely excluded. Third, our study did not categorize DMC payers, underscoring the need for closer collaboration with health insurance organizations to obtain detailed information on various payer types, including social insurance (46.1%), self-pay (26.4%), and commercial insurance (4.6%),^[39]^ which may underestimate or exaggerate patients’ choice of therapy regimens. Fourth, considering the minor effects of IgG4-RD to daily activities, indirect economic burdens, such as productivity loss and family-unit based assessments, were not included in this study,^[40]^ which might underestimate the burden of IgG4-RD for society. Fifth, our cost-effectiveness conclusions assume stable medication pricing and healthcare financing—conditions characteristic of China’s regulated pharmaceutical market. These findings may not generalize to economies with volatile drug pricing or fragmented insurance systems.

## Conclusions

Our study demonstrated the medical costs and self-estimated life quality of three common treatment regimens in IgG4-RD patients in China. While RTX demonstrates relatively positive clinical efficacy with the lowest relapse rates, its cost-effectiveness varies substantially with WTP thresholds. For healthcare systems with limited resources, GC-based regimens might be preferred options considering both clinical outcomes and medical costs. These findings support the development of treatment algorithms and policy-making improvement that balance clinical efficacy, economic sustainability, and patients’ quality of life in the management of IgG4-RD under the umbrella of various social insurance systems.
